# Letter from E. Andrews, M. D.

**Published:** 1862-08

**Authors:** E. Andrews

**Affiliations:** Memphis, Tenn.


MEMPHIS, TENN., *Aug.* 9, 1862.

MESSRS EDITORS:—Having been more or less sick with the effects of climate and soldier's life, for many weeks I have neglected you, as well as my other correspondents. Now, however, I am nearly recovered, and resume my pen, my catlin, and all my hospital implements and duties.

The military position and quiescence of the army does not favor the presentation of large surgical lists, at the present time, though the skirmishes give a continual sprinkling of cases, and now and then, a shot through the boiler of a gunboat results in a fearful catalogue of burns. A case of wounded intestine was very successfully treated by my assistant-surgeon, Dr. J. M. WOODWORTH, of Chicago. The patient received a stab with a knife, in the left iliac region, letting out the small intestines, so that they hung down externally, and were more or less covered with dirt in the scuffle. The knife, in some unexplained way, made three cuts into the intestine. Dr. WOODWORTH very carefully cleaned the viscus, and then folding in the cut edges, so as to bring the peritoneal surfaces in contact, closed the orifices with silk suture. The gut was then returned, and the external opening also closed with five stitches. The patient was put to bed, or rather to blanket, for beds are *non sunt* in the army, and a starvation regimen enforced, for ten days. The result was that he recovered, without the shadow of a bad symptom. At present, there is no tenderness on pressure, but there is an appearance as though the locality of the wound was about to become the seat of a hernia.

I mentioned, in a former letter, that at one of the fights in front of Corinth, I introduced, among the surgeons present, the idea of primary resection, hoping thereby to diminish the fearful mortality which follows military amputations. The circumstances were favorable, as the locality was elevated and healthy, and the patients were not obliged to be carried about on marches. The result went to show that no change of management can divest the cases of femurs shattered by musket balls, of fearful peril. At that battle, we resected the shattered ends of the shaft of the femur in two cases, and amputated the thigh in a third. In the superior extremity we resected two elbows and one shoulder, and amputated one forearm, making in all seven cases. The result was that all the three cases, involving the thigh, died, both the amputations and the resections terminating alike unfavorably; while in the superior extremity, the cases of amputation and the resection all lived, and did extremely well.

I am informed that Congress has passed a law, cutting down the perquisites of commissioned officers, in such a manner as to diminish the compensation of a surgeon by about three hundred dollars a-year. I have not been able to get a copy of the act, but I am informed that the diminution is made in the allowance for horses, in the following manner. Every mounted officer is entitled to have a certain number of horses, according to his rank, surgeons being supposed to have four, in time of war, and a certain allowance of feed, valued at eight dollars a-month, was furnished for each horse. But whenever the officer could dispense with a portion of the horses, without detriment to the service, he was allowed to do so, and to draw a commutation for the unused feed, in money. By many years usage in the regular army, surgeons and others were accustomed to keep only one or two horses, and draw the commutation for the others, as part of their pay. This commutation is now cut off. I fear the effect of the change will be still further to diminish the supply of medical officers, of whom the number is already very scanty.

The health of the army is excellent, the sickness being much less now than during the spring months. The number of cases under my treatment, during the last four months, is nearly as follows:

| | |
|---|---|
| April, | 388 |
| May, | 337 |
| June, | 278 |
| July, | 163 |
| Total, | 1171 |

The mortality, during this time, has been a little less than 1 per cent. of the cases, exclusive of those killed in battle.

As the season advances, the form of the sickness changes somewhat, though the cause seems to be the same, viz.: portal congestion. There is a curious relationship observable between the fevers and the class of diarrhœas. When the portal system is congested, if the patient has diarrhœa he frequently escapes fever, but if the bowels are not loose, fever results; hence the prevalence of diarrhœa is in inverse ratio to the fevers, as is shown by the following table:

| | April, | May, | June, | July. |
|---|---|---|---|---|
| Fevers, | 45 | 58 | 111 | 49 |
| Diarrhœas, | 153 | 88 | 65 | 44 |

The fevers have steadily increased, and the diarrhœas diminished until July. In the latter month, all sorts of disease, except the chronic form, underwent a marked diminution. Most of the cases of fever are remittent, though the intermittent and continuous forms are common. The typhoid fever, such as we see in Chicago, scarcely exists here at all, although there is a fictitious resemblance to it produced by the frequent occurrence of mild diarrhœa in connection with remittent fever. The diarrhœa, in such circumstances, is not dangerous, and evidently serves to mitigate the severity of the fever, so that I commonly prefer not to interfere with it very much, but let it run until I have removed the portal congestion which caused it.

Notwithstanding the supposed exemption of the negroes from the climatic diseases of the South, I am constantly seeing cases of the same fevers and diarrhœas among them, which prevail among the soldiers, and apparently of equal severity and frequency. I am inclined to think that their power of resisting Southern climatic influences is greatly overestimated, though there is doubtless something to justify the common opinion on the subject. It is positively certain, however, that they cannot endure with impunity exposure to strong malarious influences, for they are subject to all the diseases which such influences produce.

A slight examination of the operations of the medical department of the army, throws light on the enormous waste of our troops; a waste which is so great that the regiments now here cannot bring more than half their original strength to battle.

1st. There was little or no medical inspection of the recruits at enlistment. Officers, whose commissions depended on filling up their companies, cared little for the quality of the men, if they only got the number. To them the medical examination only appeared as a terrible spectre, depriving them of their recruits, and dissipating their hopes of office. Hence, in most cases, no inspection was had at all, and in others, only an inspectoral farce was enacted. For instance, when I was post-surgeon, at Camp Douglas, I received an order to repair to head-quarters, to examine a lot of recruits. Now, the army regulations require that each recruit shall be stripped naked, and inspected separately, over his whole person. Judge of my astonishment, therefore, when I was shown out upon the parade ground, in a cold winter day, with four inches of snow on the ground, and a whole regiment was paraded before me, which I was ordered to examine forthwith. I informed my superior officers, that no such inspection could be made there, as the regulations required, and that all I could do would be to pass along the lines, and pick out such obvious cases of blindness, maiming, etc., as could be seen at a glance. Accordingly, I went down the lines with my assistant, and removed six or eight bad cases. So far as I know, those recruits never had any other examination. Of course, vast numbers of men went to the field who broke down on the first hard service, and either were discharged or sent to the hospital.

2d. All the officers, both medical and others, were inexperienced in the economizing of forces, and when a man became sick, and remained so for a few weeks, they did not bear in mind the prospect of future recovery, but applied for his discharge. The higher officers, with equal prodigality, granted the request. Hence, many men were discharged for sickness, who were merely undergoing a rather protracted acclimation, and who, were they now in service, would be among the most hardy of the soldiers.

Large numbers of the sick were sent back to general hospitals in St. Louis, Mound City, Paducah, and elsewhere, who should have been kept for recovery in the regimental hospitals. The effect was just this: the surgeons in general hospitals do not feel so sensibly the necessity of keeping up the numbers of the various corps, as do field-surgeons, who are constantly associated with the regimental officers; therefore neglecting the economy of force, they seek to relieve their hospitals from overcrowding, and themselves from overwork, by procuring great numbers of discharges, and furloughs for such patients as are able to travel. Hence, vast numbers of men, who left the army with diseases of a temporary character, have never returned; and often the captains are surprised to learn that they have been discharged, when six weeks rest and treatment should have returned them to duty. The discharged men, of course, are lost; and the looseness and negligence in compelling return after furlough, renders a leave of absence as bad as a death, so far as the army is concerned.

These various causes, combined with battle and permanent sickness, have reduced the army to its present weakness. Some regiments cannot parade over 250 men under arms, and very few can present 700. The remainder, however, are hardy, well acclimated, and the officers have profited by their experience. Hereafter, the wastage by unnecessary discharges, will be very slight, and that by sickness only moderate. The actual number of deaths by sickness in the field-service of this army, has been very small; and we think that perhaps we should have actually saved many lives, had we kept more of the sick in the fresh air of the hills, and sent fewer back for the comforts of large hospitals.

Dr. JEWELL, of Attila, Ill., Demonstrator elect of Lind University, has left the army, to make preparations for removal to Chicago. He served with great credit to himself and advantage to his regiment. Dr. WOODWORTH, of Chicago, is my assistant-surgeon, and is with me at Memphis. Dr. TAYLOR, of Galesburg, is either with the army near Corinth, or else with Gen. Buell. Dr. WARDNER, of Chicago, is division-surgeon in

Gen. Davis' division, I think. I do not know his present locality, but probably somewhere between here and Chattanooga. Dr. Reilly, lately your associate editor, is, I think, with his regiment, the 45th Ill. Infantry.

A very clear illustration of the causes of intermittent fevers occurred in my regiment, at this place. Our camp here is on a dry, healthy bluff, nearly 100 feet above the river. Company A. of Chicago Light Artillery, has been for many months, the most healthy company of the regiment, the sick list seldom being over four, and those very trivial cases. Since arriving at Memphis, however, they were, after a time, suddenly attacked with a little epidemic of ague, 10 or 15 cases occurring at once. The officers suspected that the fault was in a spring from which they drew water, especially as the "Secesh," who encamped here before us, held the same spring to be so unhealthy that they put a guard over it, to prevent their men from using it. The spring, however, did not seem to me to be the cause, as it appeared to be copious, clear, and entirely free from organic matter. On a little further inspection of the ground, I found that within ten rods of the edge of the area occupied by that battery, there was a shallow depression in the soil of about one acre in extent, which a week previous, had contained stagnant rain water, being in fact a little temporary marsh. This had dried up in the hot sun, and the onset of the intermittent was simultaneous with the drying of the pool. As soon as the ground was well dried the epidemic ceased. I cautioned the officers, that in the future it would be necessary either to remove the camp, or provide for drainage of the ground.

Sunstroke has been very rare among us; and all of our diseases have been of a mild type. On the whole, we have every reason to congratulate ourselves that this climate has been very far from deadly to our armies. Yours truly,

E. ANDREWS.

